# Cerebral autoregulation: A reliable predictor of prognosis in patients receiving intravenous thrombolysis

**DOI:** 10.1111/cns.14748

**Published:** 2024-05-10

**Authors:** Zhen‐Ni Guo, Yang Qu, Zi‐Duo Shen, Jia Liu, Zhong‐Xiu Wang, Ying‐Ying Sun, Ke‐Jia Zhang, Junlei Chang, Xiang‐Kun Si, Hang Jin, Xin Sun, Yi Yang

**Affiliations:** ^1^ Stroke Center, Department of Neurology The First Hospital of Jilin University Changchun China; ^2^ Neuroscience Research Center, Department of Neurology The First Hospital of Jilin University Changchun China; ^3^ Laboratory for Engineering and Scientific Computing, Institute of Advanced Computing and Digital Engineering, Shenzhen Institute of Advanced Technology Chinese Academy of Sciences Shenzhen China; ^4^ Center for Protein and Cell‐based Drugs, Institute of Biomedicine and Biotechnology, Shenzhen Institute of Advanced Technology Chinese Academy of Sciences Shenzhen China

**Keywords:** cerebral autoregulation, intravenous thrombolysis, nomogram model, prognosis, stroke

## Abstract

**Aims:**

To investigate the characteristics of dynamic cerebral autoregulation (dCA) after intravenous thrombolysis (IVT) and assess the relationship between dCA and prognosis.

**Methods:**

Patients with unilateral acute ischemic stroke receiving IVT were prospectively enrolled; those who did not were selected as controls. All patients underwent dCA measurements, by quantifying the phase difference (PD) and gain, at 1–3 and 7–10 days after stroke onset. Simultaneously, two dCA‐based nomogram models were established to verify the predictive value of dCA for patients with mild‐to‐moderate stroke.

**Results:**

Finally, 202 patients who received IVT and 238 who did not were included. IVT was positively correlated with higher PD on days 1–3 and 7–10 after stroke onset. PD values in both sides at 1–3 days after stroke onset and in the affected side at 7–10 days after onset were independent predictors of unfavorable outcomes in patients who received IVT. Additionally, in patients with mild‐to‐moderate stroke who received IVT, the dCA‐based nomogram models significantly improved the risk predictive ability for 3‐month unfavorable outcomes.

**Conclusion:**

IVT has a positive effect on dCA in patients with acute stroke; furthermore, dCA may be useful to predict the prognosis of patients with IVT.

## Graphical abstract

Intravenous thrombolysis (IVT) has a positive effect on dynamic cerebral autoregulation (dCA) in patients with acute stroke; furthermore, dCA is an independent predictor of unfavorable outcomes in patients who received IVT and may be useful to predict the prognosis of patients with IVT.

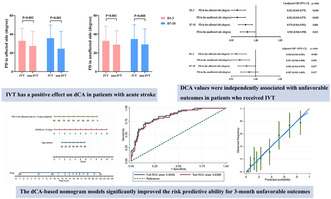

## 1 INTRODUCTION

After intravenous thrombolysis (IVT), about half of the patients fail to achieve favorable outcomes^1^; furthermore, some patients may experience hemorrhagic transformation, aggravating a poor prognosis.^2^ Thus, it is crucial to identify a reliable indicator for predicting the prognosis after IVT. Additionally, it is important to investigate possible intervention targets to improve the prognosis of these patients.

Dynamic cerebral autoregulation (dCA) can stabilize cerebral blood flow within a wide range of blood pressure fluctuations by regulating the contraction and relaxation of cerebral arteries, meaning that dCA can not only prevent hyperperfusion but also prevent infarct expansion caused by hypoperfusion.^3^, ^4^, ^5^ Thus, dCA may be an important predictor of prognosis for patients after IVT. Animal studies have reported that wild‐type tissue plasminogen activator or recombinant tissue plasminogen activator (rt‐PA) could damage cerebral autoregulation by affecting cerebrovasodilation.^6^, ^7^ However, rt‐PA‐induced recanalization or partial recanalization may minimize the ischemic core and salvage the peri‐infarct region where dCA was proven to be disturbed^8^, ^9^; thus, rt‐PA treatment may improve dCA after stroke, as found by Ma et al.^8^ Because of the inconsistency of the study results thus far, it is very important to clarify the changes in dCA in patients receiving IVT using a large sample size. Furthermore, it is also valuable to clarify whether dCA can predict the prognosis of patients after IVT or serve as a possible intervention target to improve patient outcomes.

Therefore, in the present study, to clarify the characteristics and roles of dCA in patients after IVT, we sought to (1) investigate the difference in dCA between acute stroke patients who received IVT and those who did not at 1–3 and 7–10 days after stroke onset; (2) explore the relationship between dCA and the prognosis of patients undergoing IVT; and (3) establish two dCA‐based nomogram models to verify the predictive value of dCA for patients with mild‐to‐moderate stroke receiving IVT.

## 2 MATERIALS AND METHODS

This prospective observational study was approved by the ethics committee of the First Hospital of Jilin University (2016‐294). All participants gave written informed consent, and the study was conducted in accordance with the Helsinki Declaration.

### 2.1 Participants and study design

In this study, we enrolled consecutive patients who were diagnosed with unilateral acute ischemic stroke and received rt‐PA IVT from September 2016 to March 2022 at the Stroke Center, the First Hospital of Jilin University. The inclusion criteria for the IVT group were as follows: (1) aged >18 years; (2) underwent standard rt‐PA treatment (0.9 mg/kg); (3) did not receive endovascular intervention; and (4) had modified Rankin Scale (mRS) scores ≤2 before the onset of the disease. The exclusion criteria for the IVT group were as follows: (1) with other neurological disorders, myocardial infarction, heart failure, atrial fibrillation, unstable angina within 6 months, or were classified into the cardioembolic stroke category according to the Trial of Org 10,172 in Acute Stroke Treatment (TOAST) criteria^10^ (as these may affect dCA results); (2) who could not cooperate with two instances of dCA monitoring; and (3) who had an insufficient temporal bone window on any side for insonation of the middle cerebral artery.

Patients with acute ischemic stroke who did not receive IVT were synchronously and consecutively selected as controls (non‐IVT group) for this study. The inclusion criteria for the non‐IVT group were the same as those listed in (1), (3), and (4) for the IVT group. The exclusion criteria were the same as those described for the IVT group. Additionally, during the dCA data analysis, the data that could not be analyzed due to substandard coherence function were also excluded in both the IVT and non‐IVT groups.

### 2.2 Data collection

Demographic features, vascular risk factors, and clinical information of the patients were collected. Vascular risk factors included cigarette smoking and alcohol consumption, hypertension, dyslipidemia, diabetes mellitus, previous ischemic stroke, and coronary heart disease.^11^, ^12^ Clinical information included admission systolic and diastolic blood pressure, admission heart rate, serum fasting glucose, onset to rt‐PA bolus time (IVT group), admission NIHSS score, and stroke subtypes classified according to the Trial of Org 10,172 in Acute Stroke Treatment classification (TOAST criteria).^10^ Patients with a National Institutes of Health Stroke Scale (NIHSS) ≤10 were defined as having mild‐to‐moderate stroke in this study.^13^ Clinical outcomes (mRS scores) were assessed at 90 days via telephone interviews with patients or their relatives by an examiner who was blinded to the dCA results. Favorable outcomes were defined as an mRS score of ≤2 at 90 days.

### 2.3 Evaluation of dCA

All patients underwent dCA measurements at 1–3 and 7–10 days after stroke onset. The dCA was assessed as previously reported.^3^, ^5^ Blood pressure and heart rate were measured at the brachial artery using an automatic blood pressure monitor (Omron 711, Japan) before dCA measurements, and the NIHSS score was determined simultaneously. Bilateral cerebral blood flow velocity (CBFV) in the middle cerebral artery (measured using a transcranial Doppler [MultiDop X4, DWL, Sipplingen, Germany]) and arterial blood pressure (ABP) in the digital artery (measured using a servo‐controlled plethysmograph [Finometer Model 1, FMS, Netherlands]) were recorded continuously and simultaneously for approximately 10 min. End‐tidal CO_2_ (EtCO_2_) was measured using a capnograph (MultiDop X4, DWL, Sipplingen, Germany) with a nasal cannula.

dCA was then assessed by transfer function analysis (TFA) between CBFV and ABP using MATLAB (MathWorks, Natick, MA, USA). TFA‐derived metrics included phase difference (PD), gain, and coherence function within a low frequency band (0.06–0.12 Hz).^5^, ^14^ Generally, low PD indicates impaired dCA, suggesting that CBFV passively follows changes in ABP. High gain at the same frequency band is also suggestive of compromised dCA for passively transferring the amplitude of ABP to CBFV, although it is less sensitive than phase difference.^15^ The coherence function was used to gauge linearity between CBFV and ABP, and a critical value of 0.34 (number of windows: 5; critical values of coherence: 5%) was selected as the threshold to establish the validity of the TFA estimates.^16^

### 2.4 Statistical analysis

All statistical analyses were performed using SPSS version 26.0 (IBM SPSS, Chicago, IL, USA), Stata 15.0 (Stata Corp LLC, College Station, TX, USA), and MedCalc 19.5.6 (MedCalc Software, Ostend, Belgium). Continuous variables are described as means and standard deviations, or medians and interquartile ranges in cases of skewed distribution. Categorical variables are described as frequencies and percentages. The *t*‐test, or Mann‐Whitney *U* test was used to compare group differences for continuous variables, and the χ^2^ test and Fisher's exact test were used for categorical variables to compare the difference between two groups of independent samples. The Kruskal‐Wallis test was used to compare the dCA difference in patients with different stroke subtypes classified according to the TOAST criteria. Multivariate linear models were used to explore the influence of the IVT on dCA. Variables with *p* < 0.10 from the results of the univariate analyses and those variables with established outcome‐predictive values according to the literature were selected as major covariates in the multivariable logistic regression analysis to explore the correlation between dCA and the prognosis of patients who received IVT.

To evaluate whether dCA would further increase the predictive value of conventional risk factors, three models with or without dCA were established in patients with mild‐to‐moderate stroke who received IVT to predict personalized unfavorable probability based on the results of the multivariate analysis. Variables with *p* value less than 0.10 in univariate analyses were further selected using the backward elimination method. Model discrimination was measured by calculating the area under the receiver operating characteristic curve (AUC‐ROC), and model fit was tested using the Hosmer‐Lemeshow χ^2^ test. To further assess model calibration, a calibration plot was constructed for the measurement between the observed and predicted probabilities. For the internal validation of the predictive model, we performed a 10‐fold cross‐validation. In addition, the clinical usefulness of the nomogram models was determined using decision curve analysis to quantify the net benefit. Additionally, we used AUC‐ROC, integrated discrimination improvement, and the net reclassification index to evaluate the incremental predictive value of dCA parameters beyond conventional risk factors. A comparison of the two ROC curves was based on the method described by Delong et al.^17^ A two‐sided *p* < 0.05 was considered statistically significant.

## 3 RESULTS

### 3.1 Participant characteristics

Overall, 251 patients who received rt‐PA IVT were recruited initially; 22 patients met the exclusion criteria, three patients declined to participate in the study during the follow‐up, six patients were lost to follow‐up, and dCA data of 18 patients could not be analyzed due to substandard coherence function. Initially, 270 patients in the non‐IVT group were recruited; 27 and five patients were excluded because of exclusion criteria and substandard coherence function, respectively. Finally, 202 patients in the IVT group (one patient [0.5%] had symptomatic intracerebral hemorrhage according to the European Cooperative Acute Stroke Study criteria) and 238 patients in the non‐IVT group (216 patients were not admitted to the hospital within the IVT time window, and the other 22 patients had contraindications to IVT) were analyzed. The flowchart is shown in Figure S1. The demographic and baseline characteristics of the patients included in the two groups are presented in Table S1.

### 3.2 The difference of dCA in the IVT and non‐IVT groups

PD in the IVT group 1–3 and 7–10 days after stroke onset was significantly higher compared with that of the non‐IVT group on both the affected and unaffected sides. After adjusting for confounding factors, IVT was positively correlated with higher PD both 1–3 and 7–10 days after stroke onset, suggesting that IVT had a sustained positive effect on dCA in the acute stage of ischemic stroke (Table 1).

**TABLE 1:** Comparison of dynamic cerebral autoregulation parameters between intravenous thrombolysis and non‐intravenous thrombolysis groups and the association between intravenous thrombolysis and dynamic cerebral autoregulation.

Variables	Comparison of dCA parameters between IVT and non‐IVT groups				The association between dCA parameters and IVT							
	IVT (*n* = 202)	Non‐IVT (*n* = 238)	*Z*	*p*	Unadjusted		Adjusted age + sex		Adjusted vascular risk factors^a^		Adjusted stroke data^b^	
					*β* (95%CI)	*p*	*β* (95%CI)	*p*	*β* (95%CI)	*p*	*β* (95%CI)	*p*
D 1–3												
PD in affected side (degree)	32.89 (19.26–46.85)	27.41 (11.76–43.12)	−2.134	0.002	6.342 (2.561–10.124)	<0.001	6.089 (2.381–9.815)	<0.001	6.164 (2.483–9.845)	<0.001	7.166 (3.219–11.114)	<0.001
PD in unaffected side (degree)	32.86 (19.91–48.67)	28.81 (12.34–43.68)	−3.183	<0.001	6.680 (2.942–10.418)	<0.001	6.375 (2.719–10.032)	<0.001	6.430 (2.802–10.058)	<0.001	6.476 (2.567–10.385)	<0.001
Gain in affected side (%/mmHg)	0.87 (0.68–1.20)	0.98 (0.76–1.24)	−2.026	0.043	−0.084 (−0.166 to −0.002)	0.044	−0.080 (−0.162 to 0.002)	0.055	−0.078 (−0.160 to 0.003)	0.059	−0.054 (−0.138 to 0.031)	0.214
Gain in unaffected side (%/mmHg)	0.95 (0.77–1.19)	0.98 (0.80–1.33)	−1.904	0.057	−0.104 (−0.191 to −0.016)	0.020	−0.099 (−0.186 to −0.011)	0.027	−0.094 (−0.181 to −0.008)	0.033	−0.094 (−0.184 to −0.003)	0.042
SBP (mmHg)	141.00 (123.00–160.00)	143.00 (127.00–162.00)	−0.938	0.348								
DBP (mmHg)	95.50 (82.50–108.00)	96.00 (85.00–107.00)	−0.662	0.508								
Heart rate (beats/min)	69.00 (63.00–74.00)	68.50 (63.00–75.00)	−0.401	0.688								
NIHSS score	3.00 (1.00–6.50)	3.50 (1.00–6.00)	−0.269	0.788								
End‐tidal CO_2_ (mmHg)	38.00 (35.00–42.00)	39.00 (36.00–42.00)	−1.136	0.256								
D 7–10												
PD in affected side (degree)	35.73 (18.49–49.82)	24.59 (12.07–43.01)	−3.516	<0.001	7.004 (3.071–10.937)	<0.001	6.715 (2.787–10.643)	<0.001	6.440 (2.575–10.305)	<0.001	7.900 (3.767–12.034)	<0.001
PD in unaffected side (degree)	34.77 (20.57–50.08)	29.01 (12.70–45.57)	−2.672	0.008	5.090 (1.146–9.035)	0.012	4.822 (0.901–8.744)	0.016	4.618 (0.746–8.491)	0.020	4.868 (0.751–8.985)	0.021
Gain in affected side (%/mmHg)	1.00 (0.73–1.34)	0.98 (0.73–1.26)	−0.102	0.919	−0.020 (−0.112 to 0.072)	0.675	−0.011 (−0.103 to 0.081)	0.809	−0.005 (−0.097 to 0.087)	0.923	−0.016 (−0.114 to 0.082)	0.749
Gain in unaffected side (%/mmHg)	0.99 (0.77–1.33)	1.01 (0.76–1.34)	−0.550	0.583	−0.039 (−0.131 to 0.053)	0.406	−0.030 (−0.121 to 0.062)	0.524	−0.018 (−0.109 to 0.073)	0.694	−0.042 (−0.138 to 0.054)	0.392
SBP (mmHg)	138.00 (120.00–154.50)	138.00 (123.00–154.00)	−0.255	0.798								
DBP (mmHg)	86.00 (78.00–97.00)	88.00 (78.00–98.00)	−0.981	0.327								
Heart rate (beats/min)	69.00 (63.00–74.00)	68.00 (63.00–73.00)	−0.986	0.324								
NIHSS score	3.00 (1.00–6.00)	2.00 (1.00–5.00)	−0.401	0.689								
End‐tidal CO_2_ (mmHg)	38.00 (35.00–41.00)	38.00 (35.00–41.00)	−0.483	0.629								

Abbreviation: CI, confidence interval; DBP, diastolic blood pressure; dCA, dynamic cerebral autoregulation; IVT, intravenous thrombolysis; NIHSS, National Institutes of Health Stroke Scale; PD, phase difference; SBP, systolic blood pressure; TOAST, Trial of Org 10,172 in Acute Stroke Treatment classification.

^a^ Adjusted for age, sex, and vascular risk factors (including cigarette smoking, alcohol consumption, hypertension, diabetes mellitus, dyslipidemia, previous ischemic stroke, and coronary heart disease).

^b^ Adjusted for age, gender, vascular risk factors, and clinical data (including admission SBP, admission DBP, admission heart rate, serum fasting glucose, admission NIHSS score, and TOAST).

At 1–3 days after stroke onset, gain in the affected side of the IVT group was significantly lower than that in the non‐IVT group, but multivariate linear analysis did not find a correlation between gain and IVT. On the unaffected side, there was no significant difference in gain between IVT and non‐IVT groups; however, after adjusting for confounding factors, IVT was correlated with lower gain in multivariate linear models. At 7–10 days after stroke onset, there was no significant difference in bilateral gain between the IVT and non‐IVT groups. Multivariate linear analysis also found no correlation between gain and IVT (Table 1).

We also compared the dCA difference in patients with different stroke subtypes classified according to the TOAST criteria in the IVT and non‐IVT groups (Table S2); the results suggested a remarkable impact of the TOAST classification on dCA.

### 3.3 Characteristics of dCA in the IVT group

#### 3.3.1 dCA in patients with favorable and unfavorable outcomes in the IVT group

**FIGURE 1:**
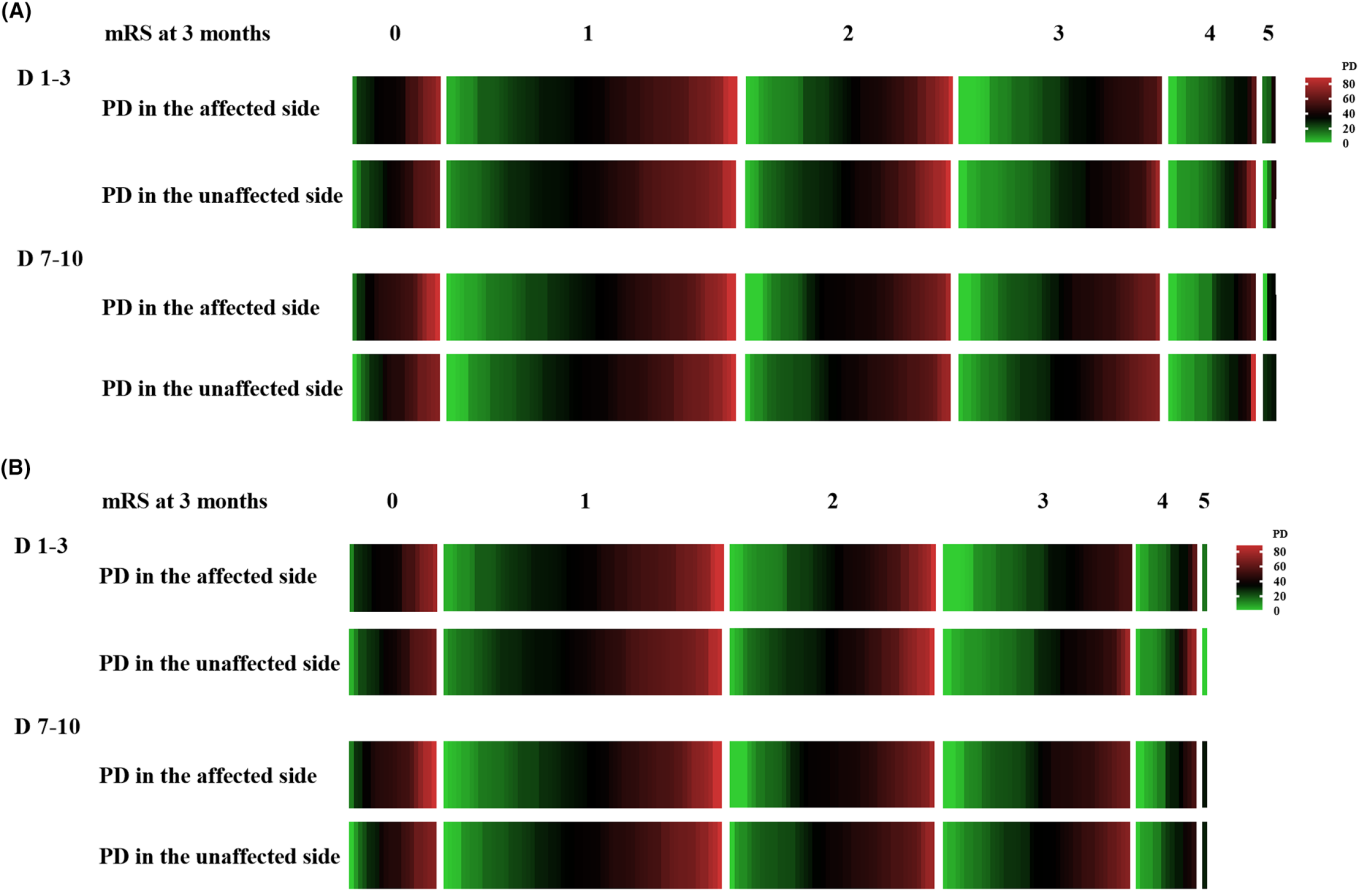
Heatmap of PD according to functional outcomes at 3 months. Values of PD measured 1–3 and 7–10 days of (A) the entire cohort of patients who received IVT and (B) patients with mild‐to‐moderate stroke who received IVT according to each mRS point at 3 months. The red is the highest and green the lowest level. IVT, intravenous thrombolysis; mRS: modified Rankin Scale; PD, phase difference.

**FIGURE 2:**
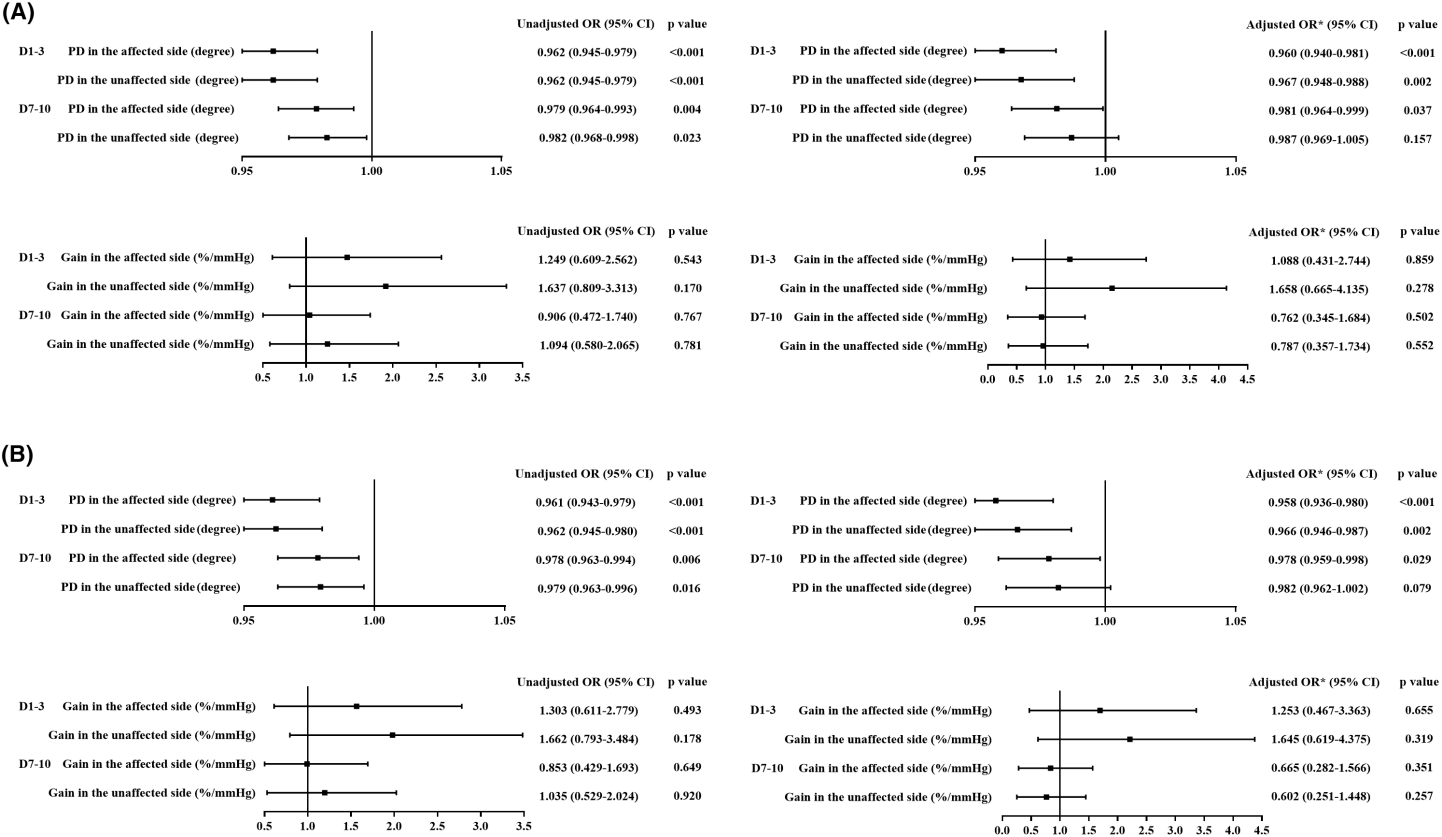
Association between dCA and 3‐month unfavorable outcome in multivariable regression analysis. Bilateral PD values 1–3 days and affected side PD values 7–10 days after onset were independent indicators of 3‐month unfavorable outcome in (A) the entire cohort of patients who received IVT and (B) patients with mild‐to‐moderate stroke who received IVT. *Adjusted for age, sex, hypertension and NIHSS score of each period, respectively. CI, confidence interval; dCA, dynamic cerebral autoregulation; IVT, intravenous thrombolysis; OR, odds ratio; PD, phase difference.

At 3 months after stroke onset, 133 (65.8%) patients had favorable outcomes, and 69 (34.2%) had unfavorable outcomes in the IVT group. PD on both affected and unaffected sides measured 1–3 and 7–10 days after IVT was significantly higher, and gain in the unaffected side at 1–3 days was significantly lower in patients with favorable outcomes than in patients with unfavorable outcomes (Figure 1, and Table 2). After adjusting for confounding factors, multiple logistic regression analysis showed that PD in both affected and unaffected sides obtained at 1–3 days and in the affected side at 7–10 days after onset were independent predictors of unfavorable clinical outcomes in acute ischemic stroke patients receiving IVT (Figure 2).

**TABLE 2:** Comparison of demographic characteristics and dynamic cerebral autoregulation parameters between patients with favorable and unfavorable outcomes in the intravenous thrombolysis group.

Variables	Favorable outcome (*n* = 133)	Unfavorable outcome (*n* = 69)	*X* ^2^/*Z*/*t*	*p*
Demographics				
Age (year)	56.92 ± 11.01	60.35 ± 8.77	−2.245	0.026
Sex (male, *n* [%])	110 (82.7%)	54 (78.3%)	0.588	0.443
Vascular risk factors				
Cigarette smoking, *n* (%)	74 (55.6%)	36 (52.2%)	0.220	0.639
Alcohol consumption, *n* (%)	67 (50.4%)	36 (52.2%)	0.059	0.808
Hypertension, *n* (%)	65 (48.9%)	46 (66.7%)	5.811	0.016
Diabetes mellitus, *n* (%)	27 (20.3%)	19 (27.5%)	1.352	0.245
Dyslipidemia, *n* (%)	104 (78.2%)	52 (75.4%)	0.207	0.649
Previous ischemic stroke, *n* (%)	20 (15.0%)	10 (14.5%)	0.011	0.918
Coronary heart disease, *n* (%)	17 (12.8%)	6 (8.7%)	0.752	0.386
Clinical data				
Admission SBP (mmHg)	157.00 (139.50–177.00)	159.00 (146.00–171.00)	−0.495	0.621
Admission DBP (mmHg)	92.00 (80.50–104.00)	96.00 (82.00–102.00)	−0.312	0.755
Admission heart rate (beats/min)	75.00 (66.00–84.00)	78.00 (64.00–90.00)	−0.498	0.619
Serum fasting glucose (mmol/L)	5.50 (4.88–6.74)	5.72 (4.86–7.34)	−0.978	0.328
Admission NIHSS score	6.00 (4.00–9.00)	8.00 (5.00–11.00)	−2.424	0.015
Onset‐to‐ rt‐PA bolus time (min)	181.00 (128.00–221.50)	188.00 (160.00–235.00)	−1.590	0.112
TOAST				
LAA	38 (28.6%)	28 (40.6%)	3.429	0.180
SAO	81 (60.9%)	33 (47.8%)		
UE	14 (10.5%)	8 (11.6%)		
dCA parameters				
D 1–3				
PD in affected side (degree)	36.62 (24.73–53.18)	24.19 (12.74–37.60)	−4.377	<0.001
PD in unaffected side (degree)	37.76 (27.37–56.43)	22.24 (11.04–40.71)	−4.618	<0.001
Gain in affected side (%/mmHg)	0.86 (0.68–1.18)	0.89 (0.68–1.24)	−0.678	0.498
Gain in unaffected side (%/mmHg)	0.89 (00.75–1.11)	1.03 (0.80–1.30)	−2.009	0.045
SBP (mmHg)	140.00 (121.50–158.50)	142.00 (126.00–167.00)	−0.749	0.454
DBP (mmHg)	97.00 (82.00–107.00)	95.00 (83.00–108.50)	−0.157	0.875
Heart rate (beats/min)	68.00 (62.00–74.00)	70.00 (64.00–74.00)	−1.021	0.307
NIHSS score	2.00 (1.00–4.00)	7.00 (5.00–9.00)	−7.569	<0.001
End‐tidal CO_2_ (mmHg)	39.00 (25.00–42.00)	38.00 (34.00–41.50)	−1.061	0.289
D 7–10				
PD in affected side (degree)	36.98 (22.97–52.04)	29.69 (13.09–44.15)	−2.740	0.006
PD in unaffected side (degree)	37.92 (22.35–52.32)	28.46 (15.22–43.54)	−2.425	0.015
Gain in affected side (%/mmHg)	1.01 (0.75–1.30)	1.00 (0.67–1.41)	−0.058	0.953
Gain in unaffected side (%/mmHg)	0.97 (0.79–1.27)	1.05 (0.72–1.36)	−0.647	0.518
SBP (mmHg)	139.00 (121.50–152.50)	138.00 (118.50–159.00)	−0.104	0.917
DBP (mmHg)	87.00 (76.50–98.00)	86.00 (80.00–94.50)	−0.166	0.868
Heart rate (beats/min)	69.00 (62.50–74.00)	69.00 (64.50–74.50)	−0.616	0.538
NIHSS score	2.00 (0.00–3.00)	6.00 (4.00–8.00)	−7.808	<0.001
End‐tidal CO_2_ (mmHg)	39.00 (34.50–41.00)	38.00 (35.00–41.50)	−0.350	0.727

Abbreviations: DBP, diastolic blood pressure; dCA, dynamic cerebral autoregulation; LAA, large artery atherosclerosis; NIHSS, National Institutes of Health Stroke Scale; PD, phase difference; rt‐PA, recombinant tissue plasminogen activator; SAO, small artery occlusion; SBP, systolic blood pressure; TOAST, Trial of Org 10,172 in Acute Stroke Treatment classification; UE, undetermined etiology.

Further, we explored the associations of EtCO_2_ levels with neurological outcomes and dCA in patients after IVT. As shown in Table 2, there was no difference in EtCO_2_ levels between patients with favorable and unfavorable outcomes. Therefore, the EtCO_2_ level was not considered a confounder. We also investigated the associations between EtCO_2_ levels and different dCA parameters at each time point. However, no significant association was found (Table S4).

#### 3.3.2 dCA in patients with mild‐to‐moderate stroke who received IVT

Since the severity of the disease (evaluated using the NIHSS score) may be the most important factor affecting the prognosis of patients with stroke, we further excluded patients with severe stroke (13 [6.4%] patients) and analyzed the predictive effect of dCA in patients with mild‐to‐moderate (NIHSS ≤ 10) stroke.^13^

One hundred eighty‐nine patients with mild‐to‐moderate stroke were analyzed. The PD trend of these patients was consistent with that of all patients (Figure 1); that is, PD in both the affected and unaffected sides obtained at 1–3 days and in the affected side at 7–10 days after onset were independently associated with unfavorable outcomes; the details are shown in Table S3 and Figure 2.

#### 3.3.3 Incremental predictive value of dCA‐based models in patients with mild‐to‐moderate stroke after IVT

**FIGURE 3:**
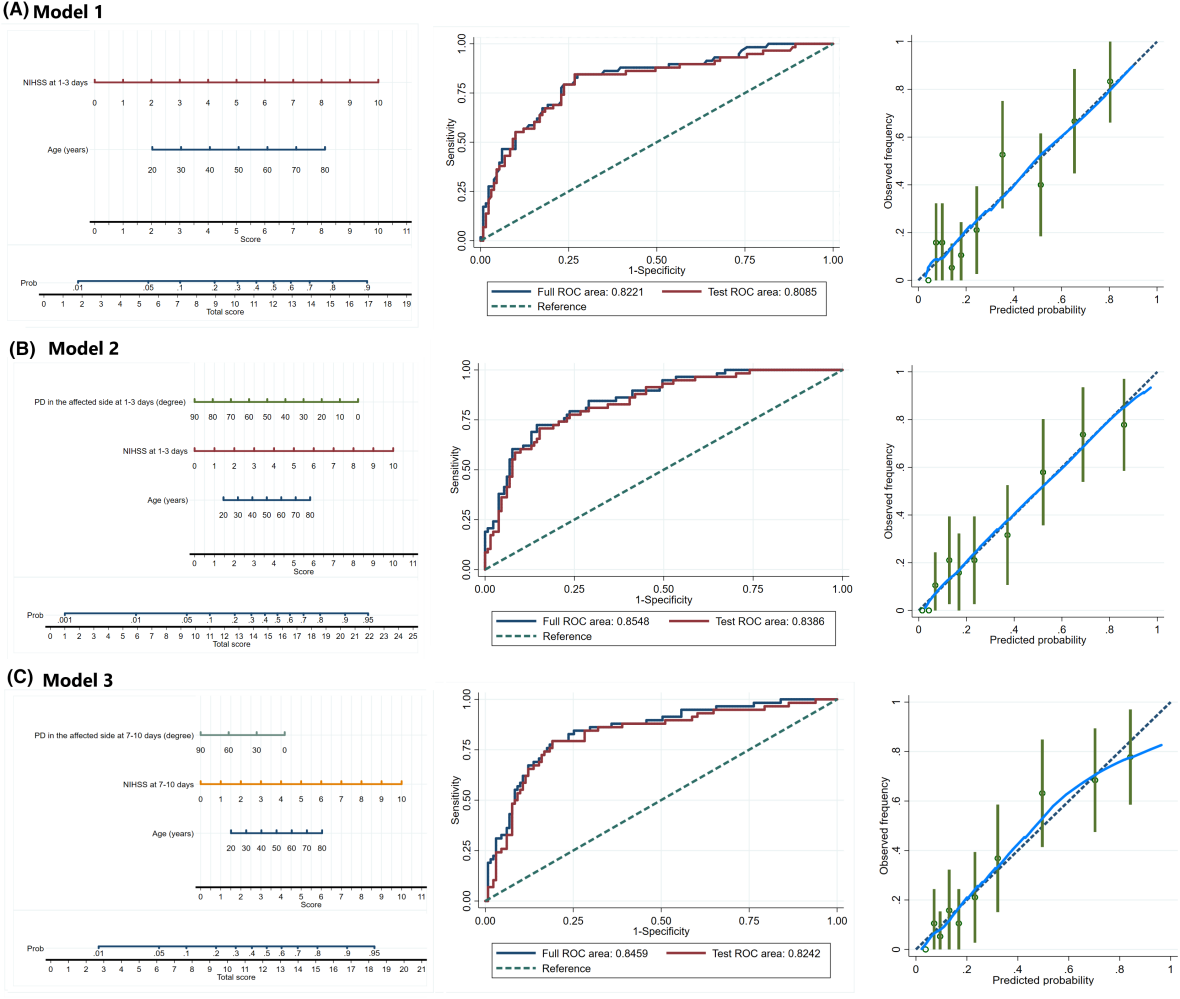
The nomogram models for predicting 3‐month unfavorable outcome. Nomograms, ROC curves, and calibration plots of (A) Model 1, (B) Model 2, and (C) Model 3. Model 1 included age and NIHSS score at 1–3 days. Model 2 included Model 1 plus affected side PD at 1–3 days. Model 3 included age, NIHSS score, and affected side PD at 7–10 days. In each model, a score was assigned for each variable by drawing a line upward from the corresponding values to the “score line.” The “total score” was calculated as the sum of the individual scores of each variable. NIHSS, National Institutes of Health Stroke Scale; PD, phase difference; ROC, receiver operating characteristic curve.

Variables whose *p* value was less than 0.10 in univariate analyses, as shown in Table S3, were further selected using the backward elimination method. Finally, Model 1 included age and NIHSS score at 1–3 days. Model 2 included Model 1 plus PD on the affected side at 1–3 days. Model 3 included age, NIHSS score, and PD on the affected side at 7–10 days. The three nomogram models are shown in Figure 3. The AUC‐ROC values of the three models were similar in the full and test models, which suggested good stability of these models, and the calibration plot suggested good predictive accuracy of the nomograms. Hosmer‐Lemeshow tests of the three models all showed *p* > 0.05, indicating that there was no statistical difference between the observed and model‐predicted probability of unfavorable outcomes (Table 3).

**TABLE 3:** Reclassification and discrimination statistics for 3‐month outcome after intravenous thrombolysis by dynamic cerebral autoregulation.

	AUC‐ROC	H‐M		NRI			IDI		
	Estimate (95% CI)	*X* ^2^	*p*	Estimate (95% CI)	*Z*	*p*	Estimate (95% CI)	*Z*	*p*
Model 1	0.822 (0.757–0.888)	9.143	0.330	Reference			Reference		
Model 2	0.855 (0.799–0.911)	4.479	0.811	48.93 (18.02–79.84)	3.103	0.002	6.55 (2.55–10.55)	3.202	0.001
Model 3	0.846 (0.785–0.907)	4.364	0.823	27.61 (−3.30 to 58.52)	1.706	0.080	2.18 (−0.52 to 4.88)	1.088	0.277

*Note*: Model 1 included age and NIHSS at 1–3 days. Model 2 included model 1 plus PD in affected side at 1–3 days. Model 3 included age, NIHSS and PD in affected side at 7–10 days.

Abbreviations: AUC‐ROC, the area under the receiver operating characteristic curve; CI, confidence interval; H‐M, Hosmer‐Lemeshow chi‐square test; IDI, Integrated discrimination improvement; NIHSS, National Institutes of Health Stroke Scale; NRI, net reclassification index; PD, phase difference.

There was no significant difference in the AUC‐ROC between Models 1 and 2 and between Models 1 and 3. However, the risk reclassification and discriminatory power of Model 2 (net reclassification index 48.93%, *p* = 0.002; integrated discrimination improvement 6.55%, *p* = 0.001) appeared to be substantially better than those of Model 1. Model 3 (net reclassification index 27.61%, *p* = 0.080; integrated discrimination improvement 2.18%, *p* = 0.227) did not show significant improvement compared with Model 1, indicating that the addition of dCA at 1–3 days significantly improved the discrimination power of the model but 7–10 days did not (Table 3). Additionally, the results of the decision curve analysis indicated that Model 2 showed a better clinical net benefit than Model 1 (Figure S2).

## 4 DISCUSSION

In this study, we found that rt‐PA IVT was positively related to dCA at 1–3 and 7–10 days after stroke onset, following adjustment for confounding factors. PD values in both the affected and unaffected sides at 1–3 days after stroke onset and in the affected side at 7–10 days after onset were independent predictors of unfavorable outcomes in patients who received IVT. Furthermore, a dCA‐based model significantly improved the risk‐predictive ability for 3‐month unfavorable outcomes in patients with mild‐to‐moderate stroke who received IVT. These results suggest that rt‐PA IVT treatment has a positive effect on dCA, and dCA has prognostic value for unfavorable outcomes in these patients. Thus, we propose that dCA could be regarded as a possible target for interventions to improve the outcomes of patients who receive IVT.

A previous study reported that low dCA in the initial hours after ischemic stroke is associated with an increased risk of hemorrhagic transformation and cerebral edema,^18^ which reflects breakthrough hyperperfusion and microvascular injury in these patients due to poor dCA. In patients who received IVT, hemorrhagic transformation is more likely to occur, which increases life‐threatening risk and aggravates the poor prognosis. Thus, maintaining a relatively good dCA is important for these patients. It has been reported that wild‐type tissue plasminogen activator can damage cerebral autoregulation in pigs.^6^ The possible mechanism is that wild‐type tissue plasminogen activator reduces the level of cyclic adenosine monophosphate in the cerebrospinal fluid, which is important to maintain vasodilation; this vasodilatory dysfunction leads to impairment of cerebral autoregulation. In addition, another study suggested that rt‐PA impairs cerebrovasodilation by activating the c‐Jun N‐terminal kinase pathway.^7^ However, these are the results of animal experiments, and the clinical situation in patients' needs to be further studied. Lam et al. conducted a self‐controlled study and found a reduction in dCA of the affected sides during and immediately after the end of IVT, but the time course of dCA changes after the end of rt‐PA and its relationship with prognosis remain unclear.^19^ Reinhard et al.^20^ reported that dCA is bilaterally preserved in minor stroke after successful rt‐PA thrombolysis, considering that there is no separate detrimental effect of rt‐PA on the cerebral autoregulatory mechanism. In our previous study, we found that rt‐PA may have a protective effect on dCA in the acute phase of stroke.^8^ Furthermore, in the present study, we selected synchronous patients with acute ischemic stroke who did not receive IVT as controls and found that dCA was significantly increased after rt‐PA IVT. This may be because the positive effect of recanalization on dCA is greater than the negative effect of rt‐PA on dCA. Notably, several clinical factors have been verified as contributing to changes in dCA in patients after ischemic stroke; carotid or intracranial arterial stenosis is one of the most widely recognized contributors. Our study also confirmed the remarkable impact of the TOAST classification on dCA (Table S2). The underlying mechanism may be that in patients with carotid or intracranial arterial stenosis, perfusion pressure is decreased, leading to vasodilation of cerebral arterioles and impairment of dCA.^21^, ^22^ In our study, to eliminate the impact of carotid or intracranial arterial stenosis on dCA, we regarded the TOAST classification as a confounding factor. Finally, we verified the independent influence of IVT on dCA.

Two previous studies reported that dCA was associated with the prognosis of patients with rt‐PA IVT. Reinhard et al.^20^ found that dCA was increasingly impaired in IVT patients with higher mRS or larger infarct volume. Nogueira et al. further compared dCA differences between patients whose symptoms were remitted (improvement of ≥4 points on NIHSS) 24–48 h after IVT and those whose were not.^23^ They found that the dCA in the latter group of patients was significantly impaired, and they speculated that dCA should be investigated as a predictor of subsequent neurological outcome. In the present study, we included a larger sample size; after considering factors that may influence prognosis in acute ischemic stroke after IVT, we found that PD values in both the affected and unaffected sides at 1–3 days after stroke onset and in the affected side at 7–10 days after onset were independent predictors of unfavorable outcomes. These results suggest that dCA can be used as an indicator to predict the prognosis of patients and that within 10 days after stroke, it can be used as the time point for dCA intervention to improve the prognosis of patients who receive IVT.

Additionally, we established two dCA‐based nomogram models, Model 2 and Model 3, to evaluate whether adding dCA would further increase the predictive ability of 3‐month unfavorable outcomes in patients with mild‐to‐moderate stroke who receive IVT. We verified substantially better discrimination power according to a significant increase in the net reclassification index and integrated discrimination improvement in Model 2. However, Model 3 did not show significant improvement compared with Model 1. Therefore, we suggest that it is better to add dCA at 1–3 days into the prognostic model of patients who receive IVT.

We believe that our study makes a significant contribution to the literature because our findings suggest that dCA can help neurologists accurately determine the prognosis of patients who receive IVT and formulate treatment strategies for these patients, such as selecting the timing of blood pressure reduction after IVT and screening appropriate patients for subsequent angioplasty. In addition, our results also show that it is feasible to explore new therapeutic strategies for IVT patients with dCA as the intervention target.^5^, ^24^

Our study did not find a significant association of EtCO_2_ levels with neurological outcomes and dCA, probably because all of the included patients in our study had normal consciousness and were asked to relax and continue breathing steadily. Therefore, the EtCO_2_ levels were sustained within the normal range and were comparable in patients in the different groups. Further studies should explore this association in patients with different consciousness levels.

However, we acknowledge that this study has several limitations. First, to enable patients to receive IVT as early as possible, dCA measurements prior to IVT were not available. Second, many patients with large vessel occlusion received endovascular treatment; therefore, they could not be included in this study, and the characteristics of dCA in these patients could not be analyzed. Third, the incremental predictive value of dCA was only explored in patients with mild‐to‐moderate stroke who received IVT. This is partly because the number of patients with severe disease was relatively small and partly because we considered the severity of the disease to be the most important factor affecting the prognosis of stroke patients, far outweighing the effect of dCA. Fourth, the number of patients with symptomatic intracerebral hemorrhage in our study was too small to analyze its relationship with dCA, so further studies are needed to explore this issue. The incidence of symptomatic intracerebral hemorrhage was low in our study owing to the following reasons: First, the stroke severity of the enrolled patients was relatively mild (mean admission NIHSS score: 6); moreover, we excluded patients with atrial fibrillation who were classified into the cardioembolic stroke category, and these patients were at risk of developing symptomatic intracerebral hemorrhage. Second, only patients who could cooperate during two instances of dCA monitoring at days 1–3 and 7–10 after stroke onset were finally included. Patients with symptomatic intracerebral hemorrhage after IVT may not cooperate with two instances of dCA monitoring because of prompt admission to the intensive care unit. Finally, the sex imbalance in this study was significant (with a male‐female ratio of 8:2), and this imbalance was considered because patients with insufficient temporal bone windows for middle cerebral artery insonation were excluded. Elderly female patients were more likely to present transtemporal window failure.^25^ Therefore, more female patients were excluded from our study, leading to the sex imbalance.

In conclusion, rt‐PA IVT treatment has a positive effect on dCA in the acute phase among stroke patients, and dCA could be used as an important indicator to predict the prognosis of patients with IVT. These results suggest that dCA could be further regarded as a possible intervention target to improve the outcome of patients who receive IVT.

## AUTHOR CONTRIBUTIONS

YY, Z‐NG, and YQ drafted the initial protocol, which was reviewed with critical revision and approval by all authors. Z‐NG and YQ wrote the first draft of the manuscript. Z‐NG, YQ, and Z‐DS did the statistical analysis. Z‐NG, YQ, Z‐DS, JL, Z‐XW, Y‐YS, K‐JZ, JC, X‐KS, HJ, and XS collected data. JL, JC, HJ, XS, and YY revised the manuscript. YY and Z‐NG acquired funding. All authors contributed to data acquisition.

## FUNDING INFORMATION

This project was supported by the National Natural Science Foundation of China (Grant No. 82271303), the Science and Technology Department of Jilin Province (YDZJ202201ZYTS677), and the Talent Reserve Program of the First Hospital of Jilin University (JDYYCB‐2023002) to Zhen‐Ni Guo, and the Norman Bethune Health Science Center of Jilin University (2022JBGS03), the Science and Technology Department of Jilin Province (YDZJ202302CXJD061, 20220303002SF), and the Jilin Provincial Key Laboratory (YDZJ202302CXJD017) to Yi Yang.

## CONFLICT OF INTEREST STATEMENT

No conflict of interest was stated.

## CONSENT TO PARTICIPATE

All participants gave written informed consent, and the study was conducted in accordance with the Helsinki Declaration.

## Supporting information


Data S1.

## Data Availability

Data is available from the authors upon request.
