# Supplementary material for: Cerebral autoregulation: A reliable predictor of prognosis in patients receiving intravenous thrombolysis
Source: CNS Neurosci Ther. 2024 May 10;30(5):e14748. doi: 10.1111/cns.14748 (PMC11086020; doi:10.1111/cns.14748)
Supplement: Supplementary file 1 — Data S1. [file CNS-30-e14748-s001.docx]

**Supplementary material**

**Cerebral autoregulation: a reliable predictor of prognosis in patients receiving intravenous thrombolysis**

**Supplementary Tables**

**Table S1. Comparison of demographic characteristics between intravenous thrombolysis and non-intravenous thrombolysis groups.**

| Variables | IVT (n=202) | Non-IVT (n=238) | X^2^/Z/t | p |
| --- | --- | --- | --- | --- |
| **Demographics** |  |  |  |  |
| Age (year) | 58.09±10.40 | 59.07±11.27 | -0.021 | 0.983 |
| Sex (male, n [%]) | 164 (81.2%) | 207 (87.0%) | 2.767 | 0.096 |
| **Vascular risk factors** |  |  |  |  |
| Cigarette smoking, n (%) | 110 (54.5%) | 138 (58.0%) | 0.553 | 0.457 |
| Alcohol consumption, n (%) | 103 (51.0%) | 117 (49.2%) | 0.146 | 0.702 |
| Hypertension, n (%) | 111 (55.0%) | 137 (57.6%) | 0.303 | 0.582 |
| Diabetes mellitus, n (%) | 46 (22.8%) | 55 (23.1%) | 0.007 | 0.933 |
| Dyslipidemia, n (%) | 156 (77.2%) | 169 (71.0%) | 2.189 | 0.139 |
| Previous ischemic stroke, n (%) | 30 (14.9%) | 53 (22.3%) | 3.928 | ***0.047*** |
| Coronary heart disease, n (%) | 23 (11.4%) | 22 (9.2%) | 0.546 | 0.460 |
| **Clinical data** |  |  |  |  |
| Admission SBP (mmHg) | 159.00 (141.00-174.00) | 157.00 (142.00-174.00) | -0.040 | 0.968 |
| Admission DBP (mmHg) | 93.00 (81.00-103.00) | 94.00 (86.00-104.00) | -1.496 | 0.135 |
| Admission heart rate (beats/min) | 75.00 (66.00-87.00) | 74.00 (67.00-80.00) | -1.537 | 0.124 |
| Serum fasting glucose (mmol/L) | 5.53 (4.89-6.98) | 5.40 (4.84-6.87) | -0.501 | 0.617 |
| Admission NIHSS score | 7.00 (4.00-10.00) | 4.00 (2.00-7.00) | -7.696 | ***<0.001*** |
| TOAST |  |  | 5.769 | 0.056 |
| LAA | 66 (32.7%) | 80 (33.6%) |  |  |
| SAO | 114 (56.4%) | 114 (47.9%) |  |  |
| UE | 22 (10.9%) | 44 (18.5%) |  |  |
| Favorable outcome | 133 (65.8%) | 167 (70.2%) | 0.943 | 0.332 |

Abbreviations: IVT: intravenous thrombolysis; SBP: systolic blood pressure; DBP: diastolic blood pressure; NIHSS: National Institutes of Health Stroke Scale; TOAST: Trial of Org 10172 in Acute Stroke Treatment classification; LAA: large artery atherosclerosis; SAO: small artery occlusion; UE: undetermined etiology.

**Table S2: The difference of dCA in different TOSTA classification.**

| **IVT group** | | | | | |
| --- | --- | --- | --- | --- | --- |
| Variables | LAA (n=66) | SAO (n=114) | UE (n=22) | H | P |
| D1-3 |  |  |  |  |  |
| PD in affected side (degree) | 24.68 (13.81-44.58) | 36.32 (24.61-52.45)^a^ | 32.03 (18.62-41.05) | 9.886 | 0.007 |
| PD in unaffected side (degree) | 29.66 (15.35-43.82) | 37.72 (22.63-54.89) | 35.33 (20.05-45.23) | 3.933 | 0.140 |
| Gain in affected side (%/mmHg) | 0.78 (0.62-1.02) | 0.89 (0.71-1.22) | 0.96 (0.81-1.33) | 8.226 | 0.016 |
| Gain in unaffected side (%/mmHg) | 0.92 (0.76-1.12) | 0.95 (0.75-1.21) | 0.98 (0.84-1.50) | 1.852 | 0.396 |
| D7-10 |  |  |  |  |  |
| PD in affected side (degree) | 23.49 (8.54-45.91) | 38.74 (24.74-51.28)^a^ | 39.40 (22.38-57.29) | 12.252 | 0.002 |
| PD in unaffected side (degree) | 28.64 (16.08-43.67) | 37.38 (22.59-50.45) | 40.71 (22.21-64.14) | 6.497 | 0.039 |
| Gain in affected side (%/mmHg) | 1.00 (0.68-1.35) | 0.97 (0.71-1.27) | 1.17 (0.93-1.46) | 5.088 | 0.079 |
| Gain in unaffected side (%/mmHg) | 1.07 (0.80-1.39) | 0.96 (0.74-1.27) | 1.18 (0.95-1.48) | 5.511 | 0.064 |
| **Non-IVT group** | | | | | |
| Variables | LAA (n=80) | SAO (n=114) | UE (n=44) | H | P |
| D1-3 |  |  |  |  |  |
| PD in affected side (degree) | 19.92 (6.49-32.35) | 32.42 (17.48-46.92)^a^ | 24.65 (3.95-38.93)^b^ | 14.135 | <0.001 |
| PD in unaffected side (degree) | 26.67 (8.10-43.82) | 31.76 (16.39-43.91) | 23.14 (9.66-39.51) | 3.801 | 0.149 |
| Gain in affected side (%/mmHg) | 0.99 (0.70-1.24) | 0.98 (0.81-1.23) | 0.93 (0.71-1.29) | 0.625 | 0.732 |
| Gain in unaffected side (%/mmHg) | 1.04 (0.81-1.38) | 0.97 (0.79-1.32) | 0.96 (0.75-1.49) | 1.048 | 0.592 |
| D7-10 |  |  |  |  |  |
| PD in affected side (degree) | 20.79 (5.55-37.13) | 30.98 (15.50-47.89)^a^ | 19.95 (14.13-32.14) | 11.883 | 0.003 |
| PD in unaffected side (degree) | 29.04 (13.93-43.85) | 31.27 (16.07-47.51) | 23.83 (8.89-45.57) | 2.681 | 0.262 |
| Gain in affected side (%/mmHg) | 0.96 (0.74-1.18) | 1.03 (0.74-1.39) | 0.98 (0.70-1.29) | 2.443 | 0.295 |
| Gain in unaffected side (%/mmHg) | 1.00 (0.76-1.35) | 1.05 (0.76-1.37) | 0.99 (0.80-1.24) | 0.158 | 0.924 |

Notes: ^a^:p<0.05 compared with LAA adjusted by Bonferroni; ^b^: p<0.05 compared with SAO adjusted by Bonferroni.

Abbreviation: IVT: intravenous thrombolysis; dCA: dynamic cerebral autoregulation; TOAST: Trial of Org 10172 in Acute Stroke Treatment classification; LAA: large artery atherosclerosis; SAO: small artery occlusion; UE: undetermined etiology; PD: phase difference.

**Table S3. Comparison of demographic characteristics and dynamic cerebral autoregulation parameters between patients with favorable and unfavorable outcomes in the intravenous thrombolysis group with mild-to-moderate stroke.**

| Variables | Favorable  outcome (n=131) | Unfavorable  outcome (n=58) | X^2^/Z/t | p |
| --- | --- | --- | --- | --- |
| **Demographics** |  |  |  |  |
| Age (year) | 56.85±11.04 | 60.45±8.92 | -2.183 | ***0.030*** |
| Sex (male, n [%]) | 108 (82.4%) | 44 (75.9%) | 1.106 | 0.293 |
| **Vascular risk factors** |  |  |  |  |
| Cigarette smoking, n (%) | 72 (55.0%) | 28 (48.3%) | 0.721 | 0.396 |
| Alcohol consumption, n (%) | 66 (50.4%) | 27 (46.6%) | 0.236 | 0.627 |
| Hypertension, n (%) | 63 (48.1%) | 39 (67.2%) | 5.934 | ***0.015*** |
| Diabetes mellitus, n (%) | 27 (20.6%) | 18 (31.0%) | 2.408 | 0.121 |
| Dyslipidemia, n (%) | 102 (77.9%) | 43 (74.1%) | 0.312 | 0.576 |
| Previous ischemic stroke, n (%) | 20 (15.3%) | 9 (15.5%) | 0.002 | 0.965 |
| Coronary heart disease, n (%) | 16 (12.2%) | 5 (8.6%) | 0.525 | 0.469 |
| **Clinical data** |  |  |  |  |
| Admission SBP (mmHg) | 157.00 (140.00-176.00) | 159.50 (146.50-171.00) | -0.737 | 0.461 |
| Admission DBP (mmHg) | 93.00 (81.00-104.00) | 95.50 (82.50-102.00) | -0.320 | 0.749 |
| Admission heart rate (beats/min) | 75.00 (66.00-83.00) | 78.00 (63.50-90.00) | -0.483 | 0.629 |
| Serum fasting glucose (mmol/L) | 5.50 (4.89-6.74) | 5.53 (4.77-7.37) | -0.592 | 0.554 |
| Admission NIHSS score | 6.00 (4.00-9.00) | 7.00 (4.00-10.00) | -1.182 | 0.237 |
| Onset-to- rt-PA bolus time (min) | 181.00 (127.00-221.00) | 188.00 (164.00-233.00) | -1.641 | 0.101 |
| TOAST |  |  | 0.975 | 0.614 |
| LAA | 38 (29.0%) | 21 (36.2%) |  |  |
| SAO | 80 (61.1%) | 32 (55.2%) |  |  |
| UE | 13 (9.9%) | 5 (8.6%) |  |  |
| **dCA parameters** |  |  |  |  |
| **D 1-3** |  |  |  |  |
| PD in affected side (degree) | 36.62 (24.75-53.22) | 24.11 (12.43-37.22) | -4.227 | ***<0.001*** |
| PD in unaffected side (degree) | 37.76 (27.42-56.55) | 21.41 (10.68-40.47) | -4.333 | ***<0.001*** |
| Gain in affected side (%/mmHg) | 0.86 (0.68-1.16) | 0.90 (0.71-1.22) | -0.884 | 0.377 |
| Gain in unaffected side (%/mmHg) | 0.89 (0.75-1.11) | 1.04 (0.82-1.29) | -2.076 | ***0.038*** |
| SBP (mmHg) | 139.00 (121.00-159.00) | 140.50 (123.00-167.00) | -0.369 | 0.712 |
| DBP (mmHg) | 97.00 (82.00-107.00) | 93.59 (82.50-109.00) | -0.066 | 0.947 |
| Heart rate (beats/min) | 68.00 (62.00-74.00) | 69.50 (64.00-74.00) | -0.796 | 0.426 |
| NIHSS score | 2.00 (1.00-4.00) | 6.00 (4.00-8.00) | -6.693 | ***<0.001*** |
| End-tidal CO_2_ (mmHg) | 38.00 (35.00-42.00) | 38.00 (34.00-41.00) | -1.095 | 0.273 |
| **D 7-10** |  |  |  |  |
| PD in affected side (degree) | 36.98 (23.12-52.28) | 29.43 (13.22-44.98) | -2.575 | ***0.010*** |
| PD in unaffected side (degree) | 37.92 (22.53-52.37) | 28.95 (15.26-42.78) | -2.406 | ***0.016*** |
| Gain in affected side (%/mmHg) | 1.01 (0.74-1.33) | 0.93 (0.62-1.40) | -0.385 | 0.700 |
| Gain in unaffected side (%/mmHg) | 0.98 (0.79-1.27) | 1.03 (0.71-1.35) | -0.368 | 0.713 |
| SBP (mmHg) | 138.00 (121.00-153.00) | 138.00 (117.50-159.00) | -0.242 | 0.809 |
| DBP (mmHg) | 87.00 (76.00-98.00) | 85.00 (80.00-94.00) | -0.074 | 0.941 |
| Heart rate (beats/min) | 69.00 (62.00-74.00) | 69.00 (65.00-74.00) | -0.592 | 0.554 |
| NIHSS score | 1.00 (0.00-3.00) | 5.00 (4.00-7.00) | -7.111 | ***<0.001*** |
| End-tidal CO_2_ (mmHg) | 39.00 (34.00-41.00) | 38.00 (34.00-41.00) | -0.436 | 0.663 |

Abbreviations: SBP: systolic blood pressure; DBP: diastolic blood pressure; NIHSS: National Institutes of Health Stroke Scale; rt-PA: recombinant tissue plasminogen activator; TOAST: Trial of Org 10172 in Acute Stroke Treatment classification; LAA: large artery atherosclerosis; SAO: small artery occlusion; UE: undetermined etiology; dCA: dynamic cerebral autoregulation; PD: phase difference.

**Table S4: The association between EtCO_2_ levels and dCA in patients after IVT.**

| Variables | Unadjusted | |  | Adjusted age+ sex | |  | Adjusted vascular risk factors^a^ | |  | Adjusted stroke data^b^ | |
| --- | --- | --- | --- | --- | --- | --- | --- | --- | --- | --- | --- |
|  | β | p |  | β | p |  | β | p |  | β | p |
| D 1-3^c^ |  |  |  |  |  |  |  |  |  |  |  |
| PD in affected side (degree) | -0.081 (-0.629 to 0.467) | 0.771 |  | -0.026 (-0.563 to 0.511) | 0.923 |  | 0.038 (-0.506 to 0.582) | 0.891 |  | 0.134 (-0.425 to 0.694) | 0.637 |
| PD in unaffected side (degree) | 0.292 (-0.254 to 0.838) | 0.293 |  | 0.361 (-0.176 to 0.897) | 0.187 |  | 0.401 (-0.144 to 0.947) | 0.149 |  | 0.513 (-0.050 to 1.075) | 0.074 |
| Gain in affected side (%/mmHg) | -0.004 (-0.014 to 0.007) | 0.514 |  | -0.004 (-0.015 to 0.006) | 0.423 |  | -0.007 (-0.018 to 0.004) | 0.222 |  | -0.004 (-0.014 to 0.007) | 0.493 |
| Gain in unaffected side (%/mmHg) | -0.002 (-0.013 to 0.009) | 0.761 |  | -0.002 (-0.013 to 0.009) | 0.666 |  | -0.005 (-0.016 to 0.006) | 0.354 |  | -0.003 (-0.013 to 0.008) | 0.594 |
| D 7-10^d^ |  |  |  |  |  |  |  |  |  |  |  |
| PD in affected side (degree) | 0.159 (-0.466 to 0.784) | 0.617 |  | 0.133 (-0.490 to 0.755) | 0.675 |  | 0.155 (-0.458 to 0.768) | 0.619 |  | 0.278 (-0.347 to 0.903) | 0.381 |
| PD in unaffected side (degree) | -0.049 (-0.642 to 0.543) | 0.870 |  | -0.079 (-0.664 to 0.506) | 0.791 |  | -0.054 (-0.631 to 0.522) | 0.853 |  | 0.050 (-0.540 to 0.641) | 0.866 |
| Gain in affected side (%/mmHg) | 0.008 (-0.006 to 0.021) | 0.259 |  | 0.008 (-0.005 to 0.022) | 0.207 |  | 0.010 (-0.004 to 0.023) | 0.160 |  | 0.012 (-0.001 to 0.026) | 0.071 |
| Gain in unaffected side (%/mmHg) | 0.001 (-0.013 to 0.013) | 0.997 |  | 0.001 (-0.013 to 0.014) | 0.910 |  | 0.003 (-0.011-0.016) | 0.707 |  | 0.005 (-0.008 to 0.019) | 0.453 |

Notes: ^a^: adjusted for age, sex and vascular risk factors (including cigarette smoking, alcohol consumption, hypertension, diabetes mellitus, dyslipidemia, previous ischemic stroke and coronary heart disease); ^b^: adjusted for age, gender, vascular risk factors and clinical data (including admission SBP, admission DBP, admission heart rate, serum fasting glucose, admission NIHSS score and TOSTA); ^c^: the association between EtCO_2_ and dCA parameters at 1-3 days after stroke onset; ^d^: the association between EtCO_2_ and dCA parameters at 7-10 days after stroke onset.

Abbreviation: EtCO_2_: End-tidal CO_2_; dCA: dynamic cerebral autoregulation; IVT: intravenous thrombolysis; PD: phase difference; SBP: systolic blood pressure; DBP: diastolic blood pressure; NIHSS: National Institutes of Health Stroke Scale; TOAST: Trial of Org 10172 in Acute Stroke Treatment classification.

**Supplementary Figure**


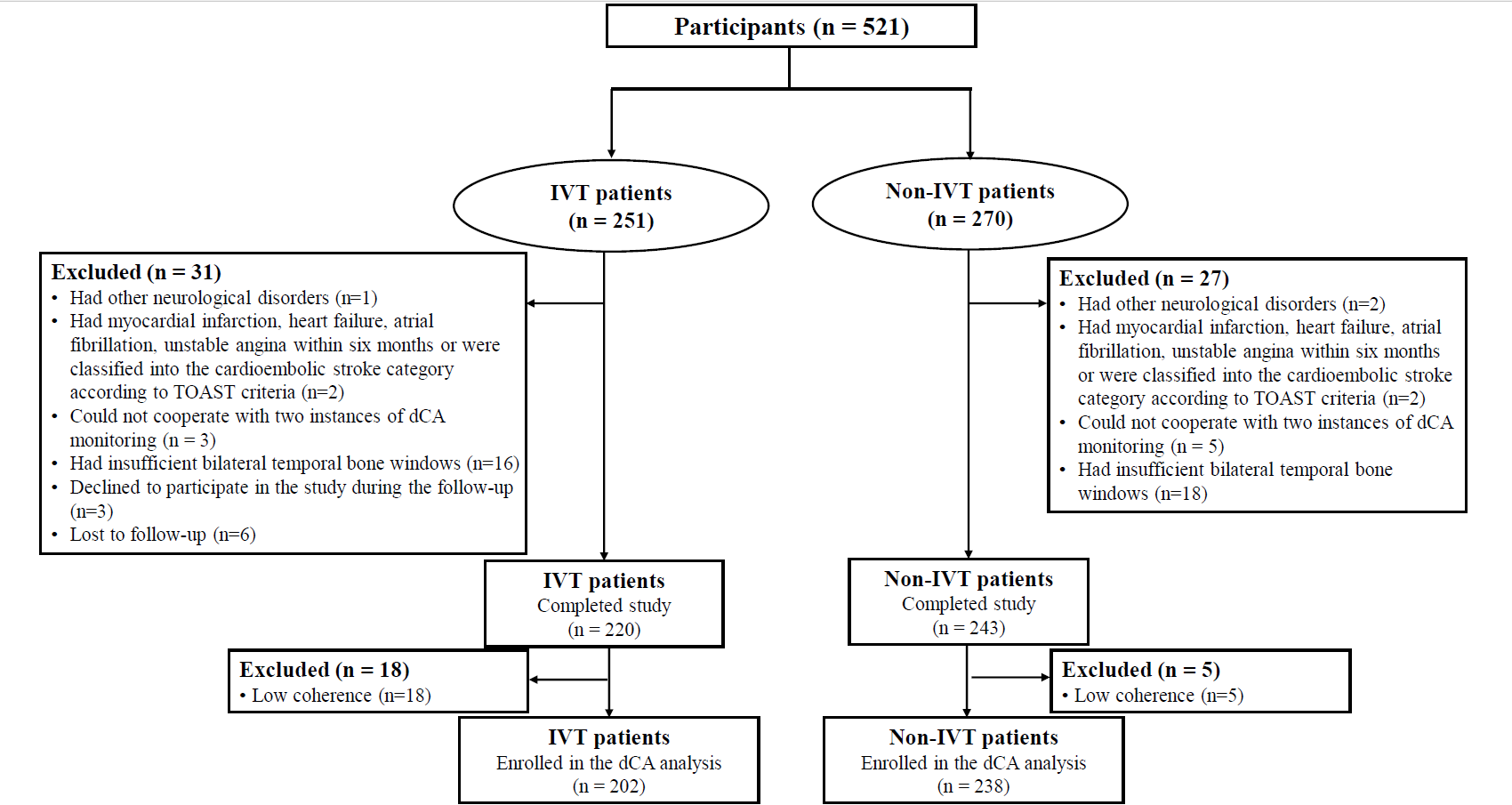


**Figure S1. The flowchart of the study.** Abbreviations: IVT: intravenous thrombolysis; TOAST: Trial of Org 10172 in Acute Stroke Treatment classification; dCA: dynamic cerebral autoregulation.

**
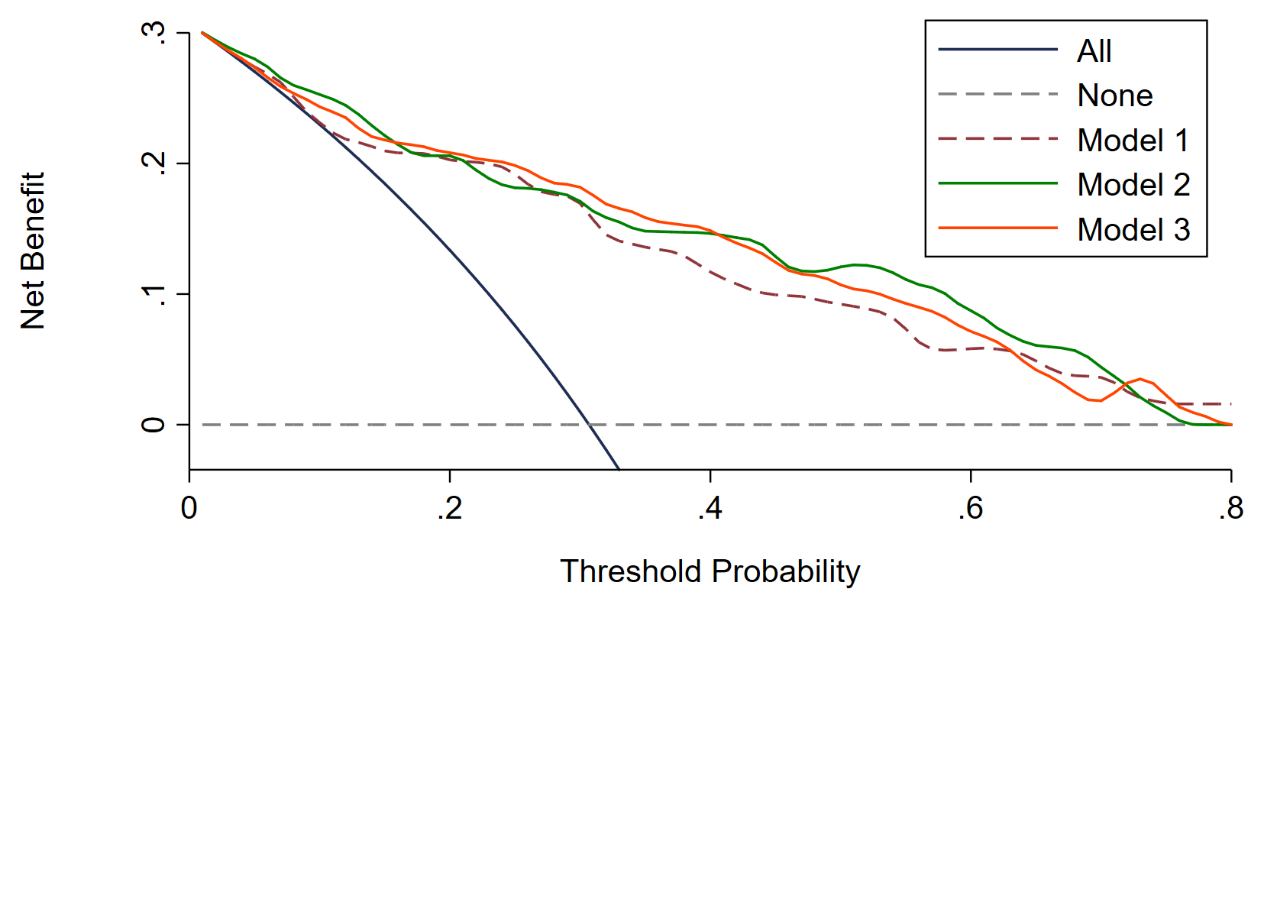
**

**Figure S2. Decision curve analysis of the nomogram models.** The black line assures that all patients develop an unfavorable outcome. The gray dotted line assures that no patients develop an unfavorable outcome. The other three lines display the net benefit of the prediction of the three models, respectively. Models 2 showed a better clinical net benefit compared to Model 1. Model 1 included age and NIHSS score at 1–3 days. Model 2 included Model 1 plus affected side PD at 1–3 days. Model 3 included age, NIHSS score and affected side PD at 7–10 days.

Abbreviations: NIHSS: National Institutes of Health Stroke Scale; PD: phase difference.
